# Inferring Causal Relationships Between Risk Factors and Outcomes from Genome-Wide Association Study Data

**DOI:** 10.1146/annurev-genom-083117-021731

**Published:** 2018-04-25

**Authors:** Stephen Burgess, Christopher N. Foley, Verena Zuber

**Affiliations:** 1 MRC Biostatistics Unit, University of Cambridge, Cambridge CB2 0SR, United Kingdom; 2 Cardiovascular Epidemiology Unit, University of Cambridge, Cambridge CB1 8RN, United Kingdom

**Keywords:** genetic epidemiology, causal inference, instrumental variable, target validation, drug discovery

## Abstract

An observational correlation between a suspected risk factor and an outcome does not necessarily imply that interventions on levels of the risk factor will have a causal impact on the outcome (correlation is not causation). If genetic variants associated with the risk factor are also associated with the outcome, then this increases the plausibility that the risk factor is a causal determinant of the outcome. However, if the genetic variants in the analysis do not have a specific biological link to the risk factor, then causal claims can be spurious. We review the Mendelian randomization paradigm for making causal inferences using genetic variants. We consider monogenic analysis, in which genetic variants are taken from a single gene region, and polygenic analysis, which includes variants from multiple regions. We focus on answering two questions: When can Mendelian randomization be used to make reliable causal inferences, and when can it be used to make relevant causal inferences?

## 1 Introduction

A modifiable exposure variable has a causal effect on an outcome if intervening on the value of the exposure leads to changes in the average value of the outcome (58). For a disease outcome, an exposure (referred to hereafter as a risk factor) is causal if intervening on the risk factor leads to changes in disease risk. Questions about causal relationships are fundamental to epidemiological research—for example, to determine disease etiology, predict the impact of a medical or public health intervention, guide drug development, advise clinical practice, or counsel on the impact of lifestyle choices.

The use of genetic variants to address questions about the causal status of risk factors by assuming that the variants are instrumental variables (IVs; see Section 2.2 for definition) is known as Mendelian randomization (28, 37). While Mendelian randomization is not the only paradigm for assessing causal relationships using genetic information, it is the most commonly used, and is the main focus of this review.

An ongoing development that is changing the way that Mendelian randomization analyses are performed is the increasing availability of summarized data from large consortia (24). Summarized data comprise beta coefficients and standard errors taken from regression (generally linear for continuous variables and logistic for binary variables) of the trait of interest on each genetic variant (22). Beta coefficients represent the average change in the trait per additional copy of the effect allele. Typically (and ideally for Mendelian randomization), these regression models additionally adjust for genetic principal components of ancestry and sometimes for age and sex, but not for further variables (11). Several consortia, such as the Global Lipids Genetics Consortium for lipid fractions (46) and the Coronary Artery Disease Genome Wide Replication and Meta-Analysis plus The Coronary Artery Disease (CARDIoGRAMplusC4D) Consortium for coronary heart disease (CHD) (33), have made these association estimates publicly available for download. A collated, queryable database of these associations can be found at http://www.phenoscanner.medschl.cam.ac.uk (85). Several other sources of these associations are also available (for example, associations estimated in UK Biobank can be found at https://sites.google.com/broadinstitute.org/ukbbgwasresults). This has facilitated the implementation of Mendelian randomization analyses, which can be conducted using these summarized data, and in particular two-sample Mendelian randomization, in which genetic associations with the risk factor and with the outcome are obtained from separate data sources (78). Two-sample Mendelian randomization is attractive because genetic associations with risk factors should be estimated in cohort or cross-sectional studies of healthy individuals (12), whereas genetic associations with disease outcomes are best estimated in case–control studies.

Even when individual-level data are available, it is often advantageous to use these to calculate the summarized associations, either to combine them with a summarized data resource to increase sample size or to consider causal estimates based on the different genetic variants to assess the IV assumptions (16). The majority of the analyses discussed in this review can be undertaken using summarized data. Software code for implementing these analyses in R based on the Mendelian-Randomization package (101) is provided in the Supplemental Appendix.

This review concentrates on answering two questions: First, when can Mendelian randomization be used to make reliable causal inferences? And second, when can it be used to make relevant causal inferences?

## 2 Monogenic Analyses: The Simple Case

We divide inquiries into causal relationships into two categories: monogenic analyses and polygenic analyses. By monogenic analyses, we mean investigations into the causal nature of risk factors using genetic variants from a single gene region. This contrasts with polygenic analyses, which include genetic variants from multiple gene regions.

### 2.1 Example: C-Reactive Protein and Coronary Heart Disease Risk

We introduce the technique of Mendelian randomization and the assumptions required using an example; technical details of all examples in the review are provided in the Supplemental Appendix. C-reactive protein (CRP) is an acute phase reactant and part of the body’s inflammatory response mechanism. Observationally, it is correlated with a multitude of traits, including cardiovascular risk factors and CHD risk (43). However, these observational associations do not imply that CRP is necessarily a causal risk factor for CHD. It could be that there are confounders—common determinants of the risk factor and the outcome—that give rise to the association. Alternatively, it could be that CRP levels increase in response to subclinical CHD, an example of reverse causation. Before spending millions of dollars developing and then validating a drug that decreases CRP levels, it would be prudent to first ensure that decreasing CRP levels will lead to reductions in CHD risk.

Genetic variants in the *CRP* gene region are associated with CRP levels (where italics indicates the gene name, and nonitalics indicates the biomarker). For each additional copy of the T allele for rs1205 (one particular variant in this region), individuals have a CRP level that is lower by an average of 0.169 standard deviations [95% confidence interval (CI) = 0.153–0.185] (32). However, the rs1205 variant is not associated with CHD risk, with an odds ratio (OR) of 0.999 (95% CI = 0.980–1.019) per additional copy of the T allele (33). Subgroups in the population with different numbers of T alleles and therefore different average levels of CRP do not have different prevalences of CHD risk. This suggests that decreasing average CRP levels in the population would not lead to reductions in CHD risk and that CRP is not a cause of CHD risk. By contrast, genetic associations with CHD risk for variants in the *IL6R* gene region (61, 63) and the *IL1RN* gene region (62) provide evidence that the interleukin-6 and interleukin-1 pathways, respectively, are implicated in CHD etiology and that these pathways are worthwhile therapeutic targets [as has now been demonstrated clinically for interleukin-1 (81)].

### 2.2 Instrumental Variable Assumptions

The risk factors CRP, interleukin-6, and interleukin-1 are excellent candidates for Mendelian randomization investigations because there are gene regions that have strong and plausibly specific links with each risk factor: the coding regions for CRP (*CRP*), interleukin-6 receptor (*IL6R*), and interleukin-1 receptor antagonist (*IL1RN*), respectively. For a genetic variant to provide a reliable test of the causal status of a risk factor, it should be associated with the risk factor but not with any confounders of the risk factor–outcome association, nor should it directly influence the outcome (41, 66). This means that subgroups of the population defined by the genetic variant differ on average systematically only with respect to the risk factor and any downstream consequences of the risk factor, and any association of a variant with the outcome is a result of the variant’s association with the risk factor.

The assumptions for a genetic variant to be an IV are (*a*) that it is relevant (the average value of the risk factor depends on the genetic variant), (*b*) that it has no association with confounders (the genetic variant is independently distributed from all confounders of the risk factor–outcome association) and (*c*) that it has no conditional association with outcome (the genetic variant is independently distributed from the outcome conditional on the confounders and the risk factor) (49). These assumptions imply the exclusion-restriction condition—namely, if the risk factor and confounders are kept constant, then intervening on the genetic variant will have no impact on the outcome (1). If the IV assumptions are satisfied, then the genetic variant can be treated like random allocation to a treatment group in a randomized controlled trial (59). Any association between the genetic variant and the outcome implies that the risk factor is a cause of the outcome in the same way as an intention-to-treat effect implies the efficacy of the treatment in a randomized trial (41). Under further parametric assumptions [sufficient conditions are that the relationships between variables are linear and that genetic associations with the risk factor and outcome do not vary with the confounder (i.e., no effect modification) (57)], a causal effect of the risk factor on the outcome can be estimated.

### 2.3 Assessing the Instrumental Variable Assumptions

The IV assumptions are unlikely to be true for all genetic variants and require a genetic variant to act like an unconfounded proxy measure of intervention on the risk factor. There are several reasons why this may not be the case (38, 47, 94). For example, the genetic variant may be pleiotropic—that is, it affects multiple risk factors on different causal pathways. Such a variant would not provide any specific information about intervention on the risk factor under analysis. Alternatively, a genetic variant may not directly affect the risk factor, but instead affect a precursor of the risk factor. If there is only one causal pathway from the genetic variant to the outcome and if this passes via the risk factor of interest, then the genetic variant would still be a valid IV (18). However, if there is an alternative pathway from the precursor of the risk factor to the outcome that does not pass via the risk factor, then again, the IV assumptions would be violated and the causal status of the risk factor cannot be reliably assessed. Other ways in which the IV assumptions may be violated have been well documented and include population stratification (the population under investigation consists of multiple subpopulations, such as different ethnic groups, meaning that genetic associations may reflect differences in allele frequencies among the subpopulations and not true biological relationships) and linkage disequilibrium (LD) (the genetic variant is correlated with another variant that influences a competing risk factor) (66).

**Figure 1:**
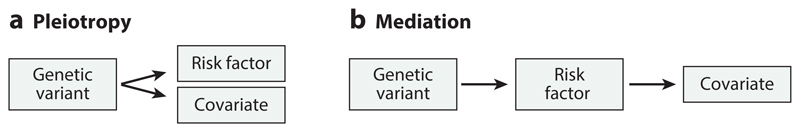
(*a*) Diagram illustrating pleiotropy (horizontal pleiotropy). The genetic variant is separately associated with the risk factor and covariate via different causal pathways. (*b*) Diagram illustrating mediation (vertical pleiotropy). The genetic variant is directly associated with the risk factor, and the association with the covariate is a downstream consequence of the risk factor.

The most straightforward way of assessing the IV assumptions is to test the association of the genetic variant with a range of potential confounders (24). However, this approach is far from foolproof. First, not all relevant confounders may be known or measured. Second, a genetic association may reflect a downstream consequence of intervention on the risk factor itself and not a pleiotropic effect (Figure 1). For example, variants in the *IL1RN* gene region linked with interleukin-1 are also associated with CRP and interleukin-6 (62). However, pharmacological intervention on the interleukin-1 receptor antagonist pathway via the drug anakinra also leads to elevated CRP and interleukin-6 levels, suggesting that these associations may reflect mediation along a single causal pathway (also known as vertical pleiotropy) rather than effects on multiple causal pathways and hence pleiotropy (also known as horizontal pleiotropy). Third, there is a multiple testing problem—one would not want to be overly conservative with testing for violations of the IV assumptions, but if large numbers of potential confounders have been measured, then some associations with the genetic variant may arise from chance alone.

Alternative ways of assessing the IV assumptions are (*a*) considering positive and negative control variables (20, 72) (for example, CRP and interleukin-6 may be considered positive controls for intervention on interleukin-1); (*b*) subsetting the population, particularly if there are subsets in the population that may have different distributions of the risk factor (for a fuller discussion, see Section 3.9); and (*c*) performing cross-ethnic analyses (if a risk factor is a cause of an outcome, then a causal effect should be evidenced across different populations, whereas patterns of LD and population stratification will differ among populations).

### 2.4 Advantages and Disadvantages of Monogenic Analyses

Among all Mendelian randomization analyses, monogenic analyses include the most reliable assessments of causal relationships. Particularly for protein risk factors, there is often a gene region that encodes either the risk factor itself or a biologically relevant factor in the causal pathway relating to the risk factor (such as the interleukin-1 receptor antagonist for interleukin-1). These gene regions have the greatest plausibility for having specific associations with these risk factors and for being reliable proxies to assess the effect of intervening on the same pathway by which the variant acts. Such investigations are also of the most value to developers of pharmaceutical agents, as the genetic variant often highlights a pathway that can be targeted by pharmacological intervention (79). Loss-of-function mutations represent potential candidate variants for monogenic Mendelian randomization investigations. However, care must be taken in the interpretation of findings (particularly quantitative findings) in this case; a loss-of-function mutation is unlikely to be a good proxy for a clinical or pharmacological intervention on the risk factor, as such interventions are unlikely to change protein function.

However, there is a sense in which performing a monogenic Mendelian randomization analysis (which is essentially a candidate gene study) is to put all of one’s eggs into a single basket. Of course, it is necessary to put the eggs into some basket, and in many cases it is better to put the eggs into a single reliable basket rather than several unreliable baskets. For example, multiple gene regions have been discovered that are associated with CRP at a genome-wide level of statistical significance, and it would be possible to conduct Mendelian randomization analyses using each of these genetic variants. However, for some of these gene regions (such as *IL6R*), the allele that is associated with increased CRP is associated with increased CHD risk, and for other gene regions (such as *APOC1*, *LEPR*, and *HNF2*), the CRP-increasing allele is associated with decreased CHD risk (16). Clearly, CRP cannot simultaneously be both protective and harmful for CHD risk. In truth, it is difficult to justify looking beyond the *CRP* gene region to assess the specific causal role of CRP. However, in other cases, combining data from multiple genetic variants associated with a single risk factor may provide convincing evidence that the risk factor is causal even when the IV assumptions cannot be clearly justified for any one variant.

## 3 Polygenic Analyses: The Difficult Case

Polygenic risk factors—risk factors that have multiple genetic determinants—include biomarkers that are more complex than proteins, such as low-density lipoprotein (LDL) cholesterol; exogenous biomarkers, such as calcium; and more complex multifactorial measures, such as blood pressure and body mass index (BMI). Mendelian randomization analyses for such risk factors are intrinsically more complex, as they must synthesize evidence from multiple genetic variants.

### 3.1 Example: Low-Density Lipoprotein Cholesterol and Coronary Heart Disease Risk

The clearest example of a plausible Mendelian randomization analysis for a polygenic risk factor is for LDL cholesterol and CHD risk. Several gene regions contain candidate IVs for LDL cholesterol; some encode a compound that is biologically relevant to LDL cholesterol, whereas others represent proxies for an existing or proposed LDL cholesterol–lowering drug.

**Figure 2:**
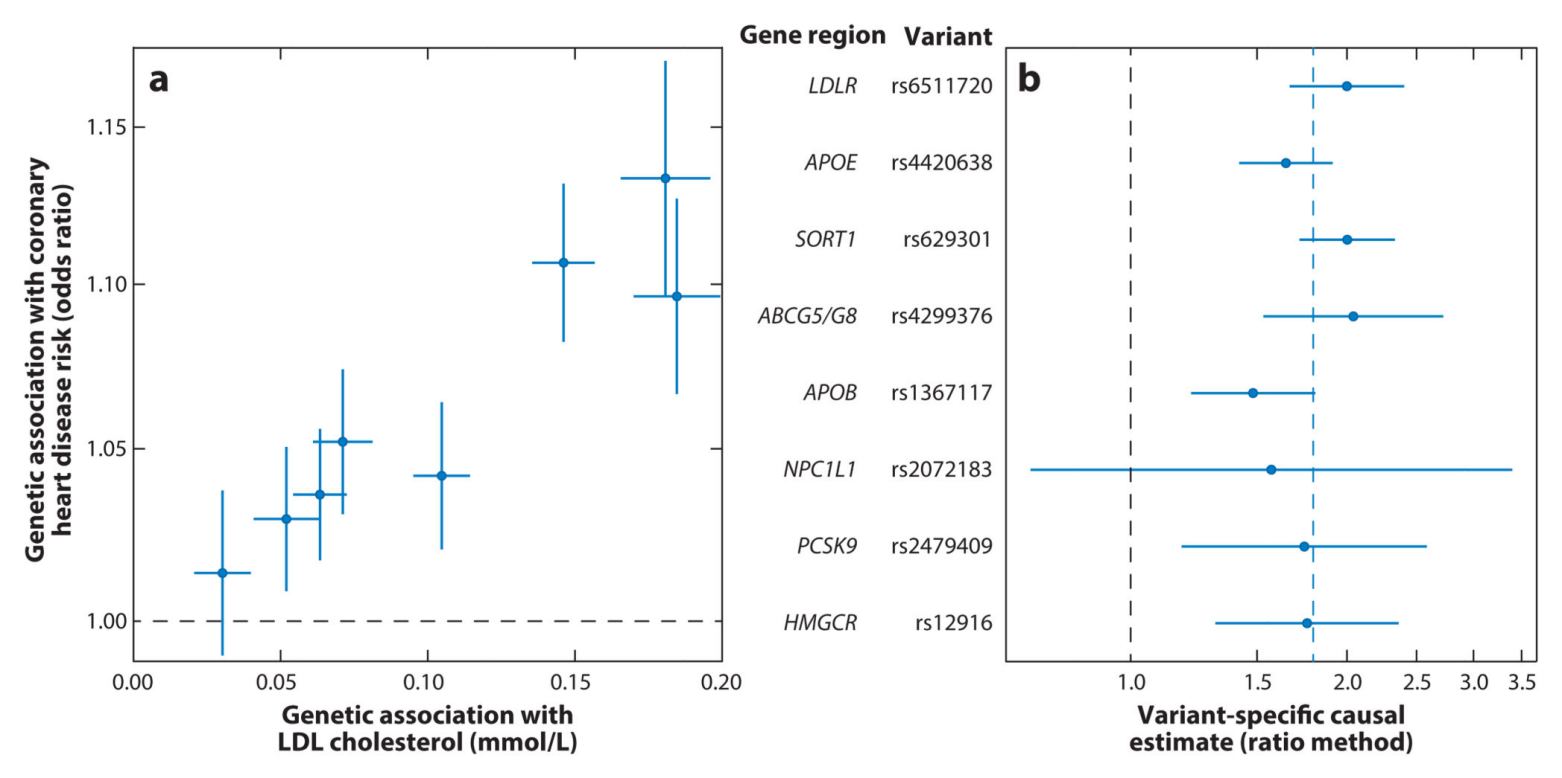
(*a*) Genetic associations with risk factor and outcome (coronary heart disease risk) for eight genetic variants that have biological links to low-density lipoprotein (LDL) cholesterol. The blue lines are 95% confidence intervals for the genetic associations (all associations are oriented to the LDL cholesterol–increasing allele); the vertical axis is plotted on a log scale. (*b*) Variant-specific causal estimates (odds ratio for coronary heart disease per 1-mmol/L increase in LDL cholesterol) from the ratio method for eight variants. The solid blue lines are 95% confidence intervals for the causal estimates; the dashed blue line is the inverse-variance weighted estimate.

Figure 2*a* shows genetic associations with CHD risk (OR per LDL cholesterol–increasing allele) taken from the CARDIoGRAMplusC4D Consortium (33) plotted against genetic associations with LDL cholesterol (per-allele changes in LDL cholesterol) taken from the Global Lipids Genetics Consortium for eight genetic variants in different gene regions (46). Not only are all of the LDL cholesterol–increasing alleles concordantly associated with increased CHD risk, but there is remarkable consistency in the causal estimates (Figure 2*b*). The gradient of the line from the origin to each point is equal to the causal estimate (assumed to be linear on the log OR scale) based on the corresponding genetic variant; this is known as the ratio estimate and represents the change in the outcome per unit change in the risk factor. Even though each variant affects LDL cholesterol via a different biological mechanism, the increase in CHD risk seems to depend only on the magnitude of change in LDL cholesterol. This consistency in the causal estimate has been observed to extend even to rare variants that have much larger genetic associations with LDL cholesterol (44).

Generally speaking, if multiple genetic variants in different gene regions all show the same direction of association with the outcome, then a causal relationship is plausible, particularly if the causal estimates based on the individual variants are all similar. As a corollary, if the causal estimates of several genetic variants are all similar, but there is one variant whose estimate differs sharply, then this genetic variant may be pleiotropic. However, it would seem unwise to make judgments about the validity of a genetic variant as an IV based solely on its associations with no reference to biological knowledge about the function of the variant; it is possible that the homogeneous variants are the invalid ones, or that the outlying variant provides important information relating to the question of causation.

### 3.2 More Complex Examples

**Figure 3:**
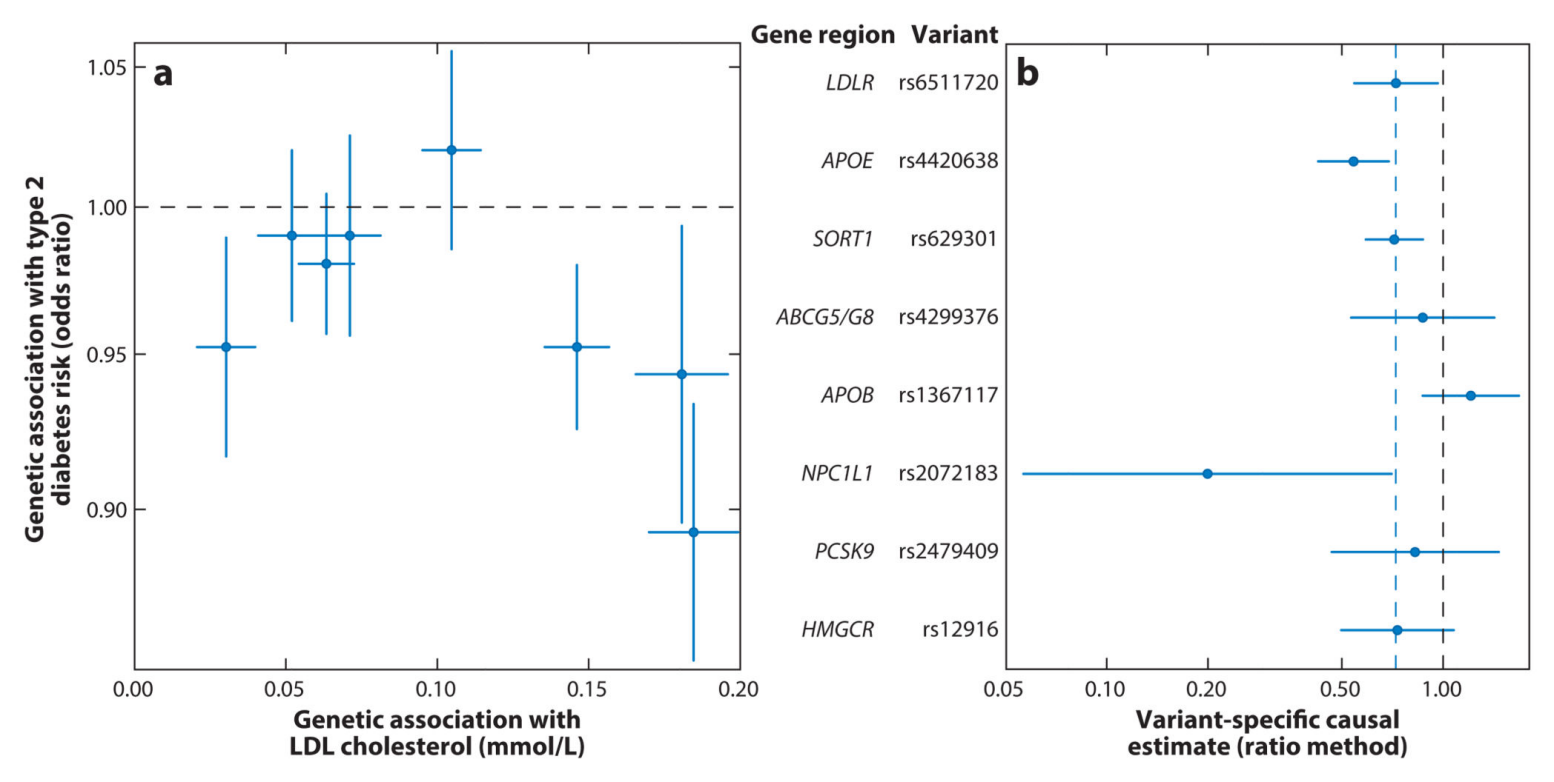
(*a*) Genetic associations with risk factor and outcome (type 2 diabetes risk) for eight genetic variants that have biological links to low-density lipoprotein (LDL) cholesterol. The blue lines are 95% confidence intervals for the genetic associations (all associations are oriented to the LDL cholesterol–increasing allele); the vertical axis is plotted on a log scale. (*b*) Variant-specific causal estimates (odds ratio for type 2 diabetes per 1-mmol/L increase in LDL cholesterol) from the ratio method for eight variants. The solid blue lines are 95% confidence intervals for the causal estimates; the dashed blue line is the inverse-variance weighted estimate.

As an example in which causal inferences are less clear, we consider associations of the same eight LDL cholesterol–related genetic variants with risk of type 2 diabetes taken from the DIAGRAM Consortium (40). For seven of the eight variants, the LDL cholesterol–increasing allele is associated with a decrease in the risk of type 2 diabetes (Figure 3*a*). The exception is the variant in the *APOB* locus. Even among those variants suggesting a protective effect of increased LDL cholesterol, the variant-specific causal estimates were not consistent in magnitude (Figure 3*b*), suggesting either mechanism-specific effects or that the effect of LDL cholesterol on type 2 diabetes risk also depends on particle size or some other aspect of lipid-related biology (73).

**Figure 4:**
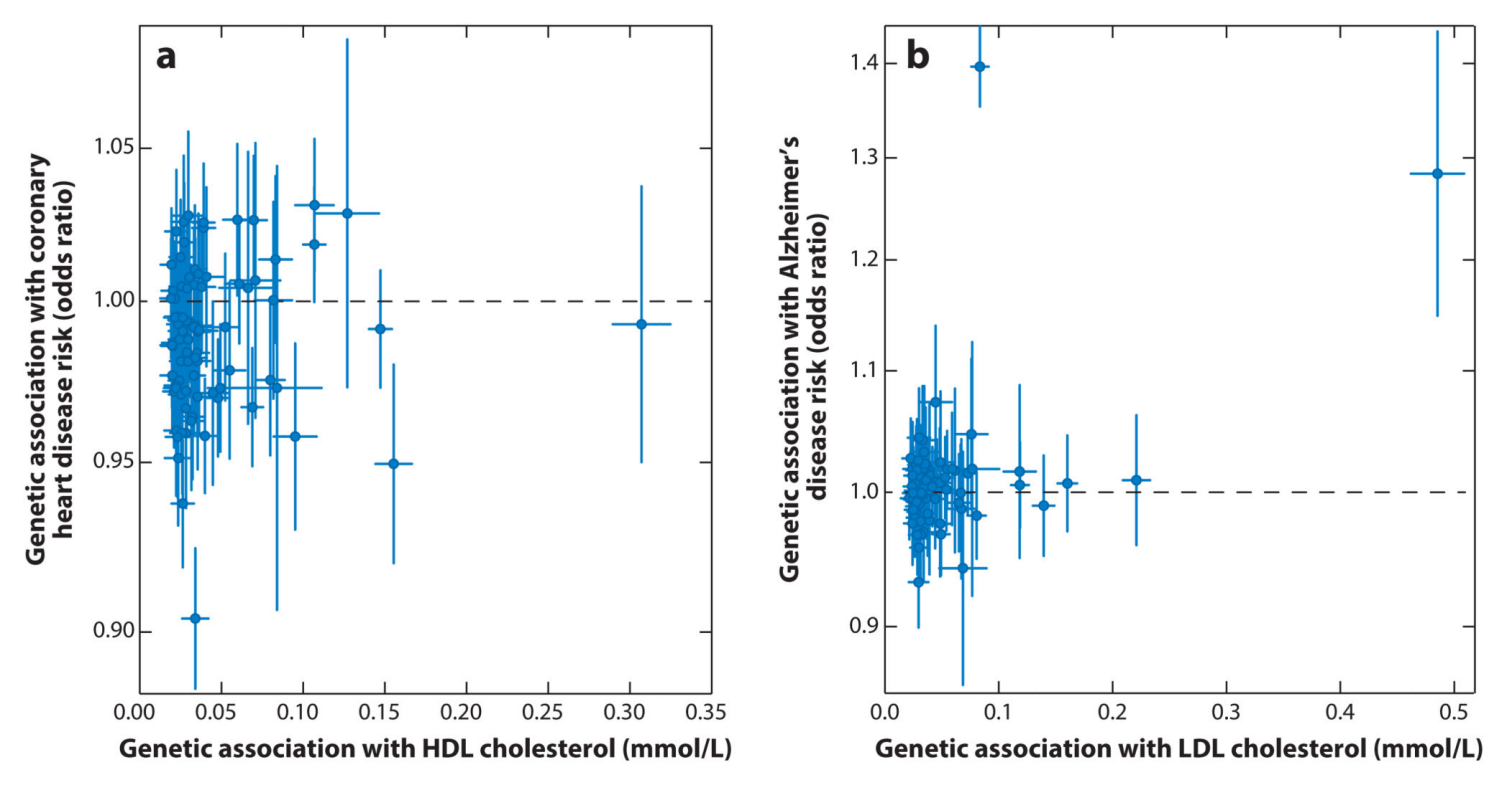
(*a*) Genetic associations with risk factor [high-density lipoprotein (HDL) cholesterol] and outcome (coronary heart disease risk) for 86 genetic variants. The blue lines are 95% confidence intervals for the genetic associations (all associations are oriented to the HDL cholesterol–increasing allele). (*b*) Genetic associations with risk factor [low-density lipoprotein (LDL) cholesterol] and outcome (Alzheimer’s disease risk) for 76 genetic variants. The blue lines are 95% confidence intervals for the genetic associations (all associations are oriented to the LDL cholesterol–increasing allele).

As examples in which causal inferences are even less clear, we consider Mendelian randomization analyses for high-density lipoprotein (HDL) cholesterol and CHD risk (Figure 4*a*), and LDL cholesterol and Alzheimer’s disease risk (Figure 4*b*). Both of the analyses include all variants previously associated with the lipid fraction risk factor at a genome-wide level of statistical significance (*p* < 5 × 10^−8^) in the Global Lipids Genetic Consortium data (46). For HDL cholesterol, this is because there are few if any variants that have specific associations with HDL cholesterol and are not also associated with other lipid fractions. In the first case, while genetic variants having less strong associations with HDL cholesterol do seem to have protective associations on average with CHD risk, variants having the strongest associations with HDL cholesterol do not also have the strongest associations with CHD risk. In the second case, while two of the genetic variants that are strongly associated with LDL cholesterol do have strong associations with Alzheimer’s disease, none of the other variants are strongly associated with Alzheimer’s disease, and the association estimates seem to be symmetrically distributed about the null. We return to these examples as we introduce the various methods.

### 3.3 Two-Stage Least Squares and the Inverse-Variance Weighted Methods

To quantitatively combine evidence from multiple genetic variants, we estimate the causal effect of the risk factor on the outcome. (The interpretation of this estimate is discussed in Section 5.) With a single genetic variant, the causal estimate (known as the ratio estimate) is obtained by dividing the beta coefficient for the association of the variant with the outcome $\left({\hat{\beta }}_{Y}\right)$ by the beta coefficient for the association of the variant with the risk factor $\left({\hat{\beta }}_{X}\right)$. When there are several IVs, the most efficient assessment of causation in terms of statistical power is the two-stage least squares method (100, chap. 15). This is undertaken by first regressing the risk factor on the genetic variants and then regressing the outcome on fitted values of the risk factor from the first-stage regression (3). When only summarized data on the per-allele genetic associations with risk factor and outcome are available, the same estimate can be obtained by the inverse-variance weighted method, which combines the association estimates (beta coefficients and standard errors) into a single estimate of the causal effect (22). This can be implemented by inverse-variance weighted meta-analysis of the ratio estimates from each genetic variant (19). Equivalently, the same estimate can be obtained from weighted linear regression of the genetic associations with the outcome (${\hat{\beta }}_{Yj}$ for genetic variant *j*) on the genetic associations with the risk factor $\left({\hat{\beta }}_{Xj}\right),$ using the reciprocals of the variances of the genetic associations with the outcome $\left[\text{se}{\left({\hat{\beta }}_{Yj}\right)}^{-2}\right]$ as weights:

1. $${\hat{\beta }}_{Yj}=\theta \;{\hat{\beta }}_{Xj}+\epsilon_{j},\;\epsilon_{j}∼𝒩\left[0,\text{se}{\left({\hat{\beta }}_{Yj}\right)}^{2}\right],$$

where *θ* is the causal effect parameter (92). If the genetic variants are correlated in their distributions, then these correlations should be accounted for in the regression model using generalized weighted least squares regression (22).

The estimate from the two-stage least squares/inverse-variance weighted method is a weighted mean of the ratio estimates based on the individual genetic variants, each of which is a consistent estimate of the causal effect when the genetic variant is a valid IV. As such, the inverse-variance weighted method generally provides a consistent estimator of the causal effect of the risk factor on the outcome only if all the genetic variants are valid IVs, and is a biased estimate if one or more of the variants is invalid.

We now consider different robust methods that can make reliable causal inferences under weaker assumptions. A robust method for Mendelian randomization is one that does not require all genetic variants to be valid IVs to give consistent estimates of a causal parameter (16). We assume for simplicity that all the genetic variants are uncorrelated in their distributions; this is usually achieved by including one genetic variant from each gene region in the analysis. We also make linearity and homogeneity assumptions as discussed elsewhere (11), in particular that all valid IVs estimate the same causal parameter (for further discussion of this point, see Section 5.1).

### 3.4 Median Estimation Methods

A conceptually straightforward method that provides consistent estimates under the assumption that at least 50% of the genetic variants are valid instruments is the simple median method (8). The median estimate is obtained by first calculating the ratio estimates based on each genetic variant and then taking the median of these estimates. With an infinite sample size, the genetic associations for each valid instrument should lie on a straight line through the origin. The gradient of this line will be the true causal effect and will correspond to the median estimate provided that at least half of the genetic variants are valid instruments (53). With a finite sample size, the simple median estimate still provides a causal estimate that is supported by the majority of the genetic variants. The median estimate is also not sensitive to genetic variants with outlying estimates—a variant contributes in the same way to an analysis regardless of whether its estimate is slightly above the median or far above it. A weighted median method has also been proposed that assigns more weight in the analysis to genetic variants that have more precise ratio estimates (8).

### 3.5 Modal Estimation Methods

A related concept is a modal estimation method. With an infinite sample size, in the case that exactly 50% of genetic variants estimate one value and 50% of the genetic variants estimate another value, it would not be possible to determine which of those values is the true causal effect. However, if the invalid genetic variants all estimate different values, then the true causal effect can be identified even when fewer than 50% of the genetic variants are valid instruments (52). If the largest group of genetic variants with the same causal estimate are the valid instruments [referred to by Hartwig et al. (54) as the zero modal pleiotropy assumption], then with an infinite sample size, the modal variant-specific ratio estimate would be the true causal effect.

With finite data, no two estimates would be exactly the same, so a modal estimate cannot be considered directly. Hartwig et al. (54) considered a kernel-density-smoothed estimate of the distribution of the variant-specific causal estimates (the ratio estimates) and took the maximum of this distribution as their modal causal estimate. Burgess et al. (31) considered a model averaging procedure that gives a mixture distribution based on the causal estimates from each subset of variants and takes the maximum of this distribution as the modal causal estimate. The model averaging procedure has several technical advantages over the kernel-smoothed approach in that it is asymptotically efficient, does not require the specification of a bandwidth parameter, and allows for uncertainty in determining which peak is the global maximum in its confidence interval. As a consequence, the method is able to identify the presence of multiple subsets of variants with similar causal estimates, suggesting the existence of multiple causal mechanisms by which the risk factor influences the outcome.

### 3.6 Regularization Methods

A further class of methods invoking a similar assumption for consistent estimation (that is, some variants are valid IVs, but we do not know which ones) uses regularization to identify valid IVs. Regularization is the use of an external condition to fit a statistical model when there are more parameters than data points. Kang et al. (65) considered a statistical model in which genetic variants influence an outcome variable not only via their associations with a risk factor but also via direct (pleiotropic) effects on the outcome. These effects cannot all be identified, as the number of parameters is equal to the number of genetic variants plus one (one pleiotropic effect parameter for each variant plus the causal effect parameter). To provide identification, they use L1 penalization (also known as the lasso) with a tuning parameter to control the total of the absolute values of all the pleiotropic effects. A related method using an adaptive lasso was considered by Windmeijer et al. (99). We here describe a simple implementation of this approach with summarized data (15). The inverse-variance weighted method (Equation 1) minimizes the weighted sum of squares:

2. $${\hat{\theta }}_{IVW}=\underset{\theta }{\text{arg}\;\text{min}}\sum_{j}\text{se}{\left({\hat{\beta }}_{Yj}\right)}^{-2}{\left({\hat{\beta }}_{Yj}-\theta \;{\hat{\beta }}_{Xj}\right)}^{2}.$$

We propose adding a separate term, *θ*_0*j*_, for each genetic variant that can be interpreted as the pleiotropic effect of variant *j*. If the term is equal to zero, this implies that the variant is a valid IV. For identification, we add an L1-penalty term:

3. $${\hat{\theta }}_{\text{LASSO},\lambda }=\underset{\theta }{\text{arg}\;\text{min}}\left[\sum_{j}\text{se}{\left({\hat{\beta }}_{Yj}\right)}^{-2}{\left({\hat{\beta }}_{Yj}-\theta_{0j}-\theta_{L}\;{\hat{\beta }}_{Xj}\right)}^{2}+\lambda \sum_{j}\left|\theta_{0j}\right|\right],$$

where *λ* is a tuning parameter and *θ_L_* is the causal parameter. When *λ* is set to zero, the parameters are not identified; when *λ* is tends to infinity, the inverse-variance weighted estimate is recovered. By considering estimates of the causal effect ${\hat{\theta }}_{\text{LASSO},\lambda }$ across a range of values for *λ*, we can compare causal inferences based on different numbers of genetic variants, with other variants being allowed to have pleiotropic effects.

Alternatively, Bayesian methods to analyze the same statistical model have been developed. These approaches use a prior for formal identification of the statistical model and to force the pleiotropic effects *θ*_0*j*_ to take values close to zero unless there is strong evidence of pleiotropy. Methods have been proposed using a horseshoe prior (6) and a spike-and-slab prior (70).

### 3.7 The MR-Egger Method

The MR-Egger method (7) was the first robust method for Mendelian randomization to become widely used and has become the de facto sensitivity analysis of choice in many people’s minds. This is somewhat unfortunate, as the assumption required for the method to give consistent estimates and valid causal inferences is not always plausible, and the MR-Egger method is quite fragile to departures from this assumption, as well as being sensitive to influential points in the regression model (30), having low power in many cases (9) and being subject to more severe weak instrument bias than other IV methods in a one-sample setting (55). However, in its defense, the MR-Egger method allows all genetic variants to be invalid IVs provided that they satisfy a different untestable assumption. Also, it is valuable to consider alternative assumptions for causal inference rather than multiple assumptions that are different flavors of “some genetic variants are valid instruments.”

In the MR-Egger method, we fit a regression model similar to that for the inverse-variance weighted method, but with an intercept term *θ*_0_:

4. $${\hat{\beta }}_{Yj}=\theta_{0}+\theta_{E}\;{\hat{\beta }}_{Xj}+\epsilon_{j},\;\epsilon_{j}∼𝒩\left[0,\text{se}{\left({\hat{\beta }}_{Yj}\right)}^{2}\right].$$

The intercept term is similar to the pleiotropic effect parameters in Equation 3, except that there is a single pleiotropy term *θ*_0_ rather than a separate term for each genetic variant. This term represents the average pleiotropic effect of a genetic variant (the expected association of a variant with the outcome if its association with the exposure is zero) (9). If all the genetic variants are valid instruments, then this term should tend to zero asymptotically. Indeed, rejection of the statistical test that this term equals zero (known as directional pleiotropy) implies that the inverse-variance weighted estimate is biased (30). This is the MR-Egger intercept test. It provides a valuable test of the validity of the set of genetic variants as IVs.

Additionally, if the genetic variants satisfy a condition known as the instrument strength independent of direct effect (InSIDE) assumption, then the MR-Egger slope estimate is a consistent estimate of the causal effect even if there is directional pleiotropy (7). The genetic associations with the outcome can be decomposed into an indirect component via the risk factor and a direct (pleiotropic) component:

5. $$\beta_{Yj}=\theta \;\beta_{Xj}+\alpha_{j}.$$

The InSIDE assumption states that the pleiotropic effects of genetic variants (*α_j_*) are independently distributed from the genetic associations with the risk factor (*β_Xj_*). This assumption would be plausible if pleiotropic effects of genetic variants were to act directly on the outcome via mechanisms unassociated with the risk factor or confounders of the risk factor–outcome association, but it is less plausible when pleiotropic effects act via related pathways or via confounders (30). The InSIDE assumption is not testable, and violation of the assumption can lead to anomalous results [for example, the estimated average pleiotropic effect can be larger than the associations of all variants with the outcome (67)—an implausible situation]. Hence, while the MR-Egger method is useful, particularly for detecting pleiotropy via the intercept test, it should not be relied on as a primary analysis method, and its findings should be weighed carefully (particularly when they differ sharply from estimates from other methods), for example, by judging whether they are particularly influenced by a single observation.

For the example of HDL cholesterol and CHD risk (Figure 4*a*), the straightforward inverse-variance method that assumes all genetic variants are valid instruments suggests that HDL cholesterol is causally protective of CHD, with an odds ratio estimate of 0.85 (95% CI = 0.76–0.95) per 1 standard deviation increase in HDL cholesterol. By contrast, the weighted median method gives OR = 0.95 (95% CI = 0.87–1.05), and the MR-Egger method gives OR = 1.10 (95% CI = 0.93–1.31) with an intercept term that differs from zero (*p* = 0.0004), suggesting no evidence for a causal relationship. All three methods agree that LDL cholesterol is a harmful risk factor for CHD risk, even when all of its genome-wide significant predictors are used in the analysis (20).

### 3.8 Multivariable Methods

Multivariable Mendelian randomization (29) is an alternative approach for causal inference that can be used when it is difficult to find genetic variants specifically and uniquely associated with particular risk factors but it is possible to find genetic variants specifically associated with a set of risk factors. For example, it is difficult to find genetic variants that are associated with HDL cholesterol but not also associated with LDL cholesterol and triglycerides (23). In multivariable Mendelian randomization, genetic variants are allowed to be associated with multiple measured risk factors, so long as they are not associated with confounders of any of the risk factor–outcome associations and do not directly affect the outcome—any genetic association with the outcome is mediated via one or more of the risk factors.

We assume that summarized data are available on the associations of genetic variants with each risk factor in turn ${\hat{\beta }}_{Xj1,}{\hat{\beta }}_{Xj2},\ldots ,{\hat{\beta }}_{XjK}$. The inverse-variance weighted method can be extended to a multivariable weighted regression model:

6. $${\hat{\beta }}_{Yj}=\theta_{1}{\hat{\beta }}_{Xj1}+\theta_{2}{\hat{\beta }}_{Xj2}+\cdots +\theta_{K}{\hat{\beta }}_{XjK}+\epsilon_{j},\;\epsilon_{j}∼𝒩\left[0,\text{se}{\left({\hat{\beta }}_{Yj}\right)}^{2}\right].$$

The parameters *θ*_1_, *θ*_2_, … , *θ_K_* represent the direct causal effects of each risk factor in turn on the outcome (the effect of varying the corresponding risk factor while keeping all other risk factors fixed). Whereas the MR-Egger method allows for unmeasured pleiotropic effects, multivariable Mendelian randomization assumes that all pleiotropic effects are measured and accounted for. These methods can be combined into a multivariable MR-Egger method that accounts for both measured and unmeasured pleiotropy (under the InSIDE assumption) by including an intercept term in Equation 6 (80).

Multivariable Mendelian randomization can also be used to consider problems of mediation in order to unravel complex etiological pathways (25). It has been used to demonstrate that the causal effect of age at menarche on breast cancer risk can be decomposed into a harmful indirect effect via decreased BMI and a protective direct effect not via BMI (39). Another area of application in which this method can be used is when there are multiple biomarkers influenced by several genetic variants in a single region. For example, multivariable Mendelian randomization suggested that genetic associations with atopic dermatitis of variants in the *IL1RL1*–*IL18R1* locus on chromosome 2 are driven by IL18R1 and IL1RL2 rather than by IL1RL1 (87).

### 3.9 Interactions and Subsetting

Another approach for assessing the validity of causal inferences is exploiting subsets within the data set. For example, in East Asian societies, women tend not to drink alcohol for cultural reasons. Hence, if alcohol is a cause of a disease outcome, then genetic variants that influence alcohol metabolism should be associated with the outcome in men but not in women, as there is no mechanism by which the genetic variants would affect the outcome in individuals who have zero exposure to alcohol (34). If the same genetic associations with the outcome were present in both men and women, this would suggest that they are driven not by alcohol consumption, but rather by a pleiotropic mechanism (93).

Additionally, stronger genetic associations with esophageal cancer risk have been observed in populations that drink heavily, and no association has been observed in men who abstain from alcohol (69). Some care is needed here, as the decision to abstain from alcohol is self-determined, and hence conditioning on abstinence may lead to selection bias (56). However, in this case the differences between the genetic associations in drinkers and nondrinkers are so extreme that it is highly unlikely that they are explained by selection bias alone. The conditions that the subsets must satisfy have been provided; ideally, the genetic associations with the risk factor should be present in one subset but not in the other, but any pleiotropic effects of the genetic variants should be identical across subsets (93).

This idea has been extended to interactions with a continuous variable under the name Slichter regression (84). Similar conditions are required—genetic associations with the risk factor should depend on the interacting variable, but any pleiotropic effects should not be subject to modification by the interacting variable.

### 3.10 Practical Advice

In practice, we would encourage investigators to plot the genetic associations with the risk factor against those with the outcome (as in Figures 2*a*, 3*a*, and 4*a,b*) as a routine part of polygenic Mendelian randomization investigations. This provides a visual check for heterogeneity and enables both investigators and readers to judge whether there is general consistency in genetic associations and whether any claimed causal effect is evidenced by all of the genetic variants or just a subset of them. Formal tests for heterogeneity are also available (10, 48). Other plots that have been suggested for investigating pleiotropy include forest plots (68) and funnel plots (86), taken from the meta-analysis literature.

While we have presented a long list of robust methods for Mendelian randomization, we would not expect every Mendelian randomization investigation to include all of these analyses. Indeed, we would encourage investigators to think carefully about which sensitivity analyses would be most appropriate given their particular investigation. For example, if there are genetic variants that are likely to be outliers or influential points (such as variants in the *APOE* gene region for Alzheimer’s disease or in the *FTO* gene region for BMI), then an outlier-robust approach may be more appropriate than the MR-Egger method.

As an example of how robust methods can reveal inconsistencies in a Mendelian randomization investigation, we consider the investigation into the causal effect of LDL cholesterol on Alzheimer’s disease using 380 genetic variants reported by Benn et al. (4). Causal estimates represent ORs per 1-mmol/L decrease in LDL cholesterol. The authors initially reported a highly significant association based on the inverse-variance weighted method (OR = 0.83, 95% CI = 0.75–0.92) and the MR-Egger method (OR = 0.64, 95% CI = 0.52–0.79). However, the funnel plot of the variant-specific causal estimates plotted against their precisions revealed variants with extreme outlying causal estimates, and the weighted median method gave a much different estimate (OR = 0.97, 95% CI = 0.91–1.02). Further investigation showed that the variants suggesting a strong causal effect were all located in and around the *APOE* gene region, a known risk factor for Alzheimer’s disease (note that the two variants strongly associated with Alzheimer’s disease in Figure 4*b* are both in the *APOE* gene region). Analyses excluding these variants suggested no causal effect in either the inverse-variance weighted method (OR = 0.98, 95% CI = 0.93–1.03) or the MR-Egger method (OR = 0.98, 95% CI = 0.87–1.09) (4; see the “rapid response” written by the authors).

## 4 Other Approaches for Investigating Relationships between Traits with Genome-Wide Association Study Data

Mendelian randomization is not the only methodological approach for considering relationships between risk factors and outcomes using genetic data, although it is the only approach that directly assesses the causal effect of a risk factor on an outcome. Other approaches include colocalization and LD score regression.

### 4.1 Fine Mapping and Colocalization

Fine mapping is a statistical approach to find genetic variants that causally affect a trait (5). We note that a causal genetic variant is not required in a Mendelian randomization investigation—the first IV assumption only requires a genetic variant to divide the population into groups with different average levels of the risk factor (57).

Colocalization methods take a single region of the chromosome that is associated with two traits and ask whether the same genetic variants are driving the associations with both traits (82). As such, it is an extension of fine mapping to multiple traits. If the same genetic variants (or variant) that give rise to the association with trait A also give rise to the association with trait B, then the same biological mechanism is likely to be responsible for both associations. This does not necessarily mean that trait A causes trait B (or vice versa), as it may be that a trait C causes both A and B. However, even in this case, colocalization implies a shared etiology between traits A and B.

As an example, colocalization showed that a genome-wide association study hit in the *CTSH* gene region drove genetic associations with both type 1 diabetes and narcolepsy (50). The same variant was also associated with gene expression but localized to monocytes and not to B cells. These results demonstrate the utility of genetic association data for highlighting causal cell types and potential druggable mechanisms.

**Figure 5:**
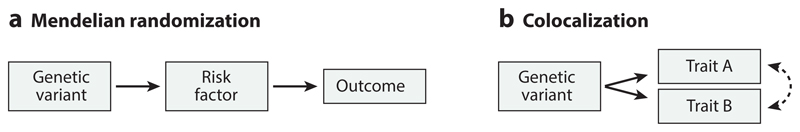
Schematic diagrams illustrating different assumptions made in (*a*) Mendelian randomization (in which the genetic variant is assumed to associate directly with the risk factor and with the outcome only via the risk factor) and (*b*) colocalization (in which the genetic variant is allowed to associate with both traits directly, and causal effects may occur between the traits in either direction or not at all).

Figure 5 illustrates differences between the assumptions made in Mendelian randomization and in colocalization. In some cases, the statistical methodology used in the two approaches will be identical (97); differences in the conclusions (for example, concluding that a risk factor is a cause of the outcome) are conceptual and arise solely from different prior assumptions.

Colocalization methods are useful when a particular section of the chromosome has multiple genes in close proximity (45, 60). It may be that genetic variants that are associated with a risk factor also show an association with a disease outcome. This would lead to a positive finding in a Mendelian randomization analysis. However, if the colocalization analysis showed that the peaks of the associations were being driven by different variants, then this would suggest that the risk factor is not the relevant causal agent. This could result from LD between two functional variants. It could also be that the relevant measure of the risk factor has not been identified (for example, suppose that the risk factor is not LDL cholesterol concentration but rather a measure of LDL particle size).

Overall, colocalization focuses on a different causal question than does Mendelian randomization, one that focuses on a single gene region rather than considering the totality of evidence for a relationship between a risk factor and an outcome across multiple gene regions. However, the more detailed interrogation of a single gene region performed in the colocalization method can provide additional evidence to link a risk factor to an outcome. Colocalization can also be performed between two disease outcomes, to assess whether the diseases have a common etiological link even in the absence of a hypothesized mechanism or risk factor for that link.

### 4.2 Linkage Disequilibrium Score Regression

There are two forms of LD score regression: with a single trait (14) and with a pair of traits (13). The aim of the method is to provide estimates of heritability (shared heritability for two traits) that are unaffected by population stratification. Both approaches make use of LD scores. These are obtained for each variant in turn by taking a window about the target variant, calculating the squared correlation for each variant in this window with the target variant, and summing these squared correlations. A genetic variant with a high LD score is a variant with a high degree of “friendliness”—it has many nearby variants in high LD with it. If biologically relevant genetic variants are uniformly distributed across the whole genome, then a genetic variant with a high LD score is more likely to be correlated with a biologically relevant variant and hence is more likely to be associated with phenotypic traits, including risk factors and outcomes. By contrast, genetic variants that are subject to population stratification would not be more or less likely to have high LD scores, so any genetic associations resulting from population stratification would be independent of LD score.

With a single trait, LD score regression is implemented by regression of the chi-squared statistics for the genetic associations with the trait against the LD scores for genetic variants across the whole genome (14). The intercept term is interpreted as confounding (although by “confounding,” the authors are referring to population stratification and cryptic relatedness between individuals rather than conventional epidemiological confounding), and the slope coefficient is interpreted as a revised estimate of heritability (narrow-sense heritability) that excludes population stratification. Weights are used in the regression model to correct for correlations between variants and for the varying precisions of the chi-squared statistics. With two traits, cross-trait LD score regression is implemented by multiplying the *z* statistic for the genetic association with trait 1 by the *z* statistic for the genetic association with trait 2 and then regressing this product of statistics against the LD scores (13). The slope coefficient (representing shared heritability or genetic correlation) is large when the same genetic variants predict both of the traits. Regression on the LD score should ensure that estimates of heritability are driven by biologically relevant variants rather than variants subject to population stratification. However, an implicit assumption in the method that variants contribute equally to heritability estimates may be violated in realistic settings, leading to bias and incorrect standard errors (83).

LD score regression differs from Mendelian randomization in several ways. First, LD score regression is not only a polygenic method but a massively polygenic method, using genetic variants from the whole genome. Second, LD score regression is symmetric in its two traits, whereas Mendelian randomization assesses the effect of the risk factor on the outcome. It does not seek to model one trait as a function of the other but rather uses the LD score as the dependent variable in the regression model.

However, there are also connections between the approaches. In the MR-Egger method, the intercept term in the regression model is viewed as a nuisance parameter relating to the degree of pleiotropy, and the slope parameter is an estimate of the causal effect. In LD score regression, the intercept term is again a nuisance parameter, and the slope parameter is of interest. This suggests that a version of the InSIDE assumption is required by the LD score regression paper (no correlation between the residual terms in the LD score regression equation and the LD scores). The problem of low power in the MR-Egger method can be sidestepped in cross-trait LD score regression by specifying rather than estimating the intercept term.

A criticism of LD score regression is that every analysis for each pair of traits uses the same LD scores as the dependent variable in the regression model (and as LD scores have been pre-computed by its proponents, literally the same LD scores are used in the majority of applied analyses). This means that any influential points in the regression will affect not only one LD score regression analysis but all such analyses. LD scores are also likely to be a weak instrument in the language of Mendelian randomization, as they will explain only a small proportion of variance in the dependent variable. Additionally, owing to the scale of the data, it is not possible to provide a visual representation of an LD score regression analysis. Standard regression diagnostics are rarely, if ever, performed. Finally, results from LD score regression are not always consistent with known causal relationships; for example, the method did not find evidence for a genetic correlation between LDL cholesterol and CHD risk that survived a multiple testing correction (13). The method has utility in mapping the genetic distance between related phenotypes, such as determining how closely related different psychiatric disorders are in terms of their genetic predictors (36). However, the reliance of the method on numerous linearity and independence assumptions, incorrect weighting in the linear regression model (correct weights would require computation of the Cholesky decomposition of a matrix with dimension equal to the number of genetic variants in the model—misspecified weights are recommended for use in practice), and a lack of validation against known causal relationships mean that results from the method should not be treated too seriously as an assessment of causality.

## 5 The Relevance of Causal Inferences and Estimates from Mendelian Randomization

While Mendelian randomization aims to address the question of whether a risk factor is a cause of an outcome, in reality this is not a well-defined question. For example, in answering the question “Would lowering BMI reduce cardiovascular mortality?” several further questions arise: How is BMI proposed to be lowered? By increasing metabolism? By suppressing appetite? How long is BMI proposed to be lowered for? To whom will the intervention be applied? To answer the question “Is high BMI a cause of increased cardiovascular mortality?” one must first pose the question in a precise way.

A Mendelian randomization investigation compares subgroups in the population defined by their genetic variants. As such, it is analogous to a randomized controlled trial, but one in which the allocation to a “treatment group” is determined at conception. In a randomized trial, typically the treatment that is assessed in the trial is also the one that is offered to patients. However, in Mendelian randomization, the intervention that is assessed is changing an individual’s genotype at conception, whereas the proposed treatment is to change the value of the risk factor by a different mechanism (that is, not by altering their genes) and at a later time (usually in adulthood) (88). As such, Mendelian randomization estimates (and, equivalently, assessments of causal relationships) typically represent the impact of a long-term (or even lifelong) intervention in the risk factor by a small amount in a primary prevention context (17). In many cases, the Mendelian randomization investigation does address a relevant causal question. For example, one may be interested in whether a CRP-reducing agent should be included in a polypill that is taken every day to reduce mortality risk in healthy individuals. However, a Mendelian randomization investigation would be less relevant for judging whether short-term highly elevated CRP levels should be targeted for intervention in individuals displaying acute coronary symptoms.

### 5.1 Heterogeneity and Pleiotropy

For a complex multifactorial risk factor such as BMI, there are many mechanisms by which the risk factor could be lowered and many genetic variants associated with BMI that influence the risk factor via different biological pathways. It is therefore entirely possible that some, but not all, genetic variants associated with the risk factor are also associated with the outcome, or that all variants are associated with the outcome but the causal effects estimated by different variants differ. This is both a blessing and a curse for Mendelian randomization. On the positive side, this can be used to identify which aspects of a complex risk factor influence an outcome and hence may help identify effective mechanisms for reducing disease risk (98). On the negative side, the methods above rely on homogeneity of the causal estimates based on different genetic variants and interpret deviation from this as pleiotropy.

Even if there is heterogeneity in causal estimates, the ratio estimate based on each genetic variant is still a valid test of the causal null hypothesis (that is, under the IV assumptions, it will only differ from zero if the risk factor is a cause of the outcome) (22). Hence, the inverse-variance weighted method still provides a valid test of the causal null hypothesis, as do the median-based and mode-based methods. However, other methods, such as MR-Egger, are more sensitive to these parametric assumptions. One practical solution is the recommendation to use random-effects models in estimation, as these models translate heterogeneity between variant-specific causal estimates into wider confidence intervals (11). Heterogeneous points should be investigated carefully to assess whether there is any difference in how the genetic variant influences the risk factor that may give rise to the heterogeneity or whether it is likely to be a result of pleiotropy.

### 5.2 Time-Dependent Causal Effects

Some methodological development has been undertaken to try to perform IV analysis with a time-to-event outcome (74, 91). While it is possible to assess the causal null hypothesis with a time-to-event outcome [by assessing the association between the genetic variant(s) and outcome], any causal estimates are unlikely to be reliable. Indeed, it would seem to be impossible to make any detailed judgment about the timing of causal effects using genetic variants, unless one had specific temporal knowledge about the action of the genetic variants on the risk factor. For example, if increased BMI leads to increased cardiovascular mortality, then associations of genetic variants would be identical if increased childhood BMI led to increased cardiovascular mortality, or if the causal risk factor were instead increased adolescent BMI or increased BMI in early adulthood—assuming that the genetic variant is associated with increased BMI for the whole life course. These scenarios could be distinguished only by identifying and comparing associations for particular genetic variants that increase BMI in childhood, those that increase BMI in adolescence, and so on.

Additionally, it is not possible (without additional information) to judge the viscosity of a risk factor. By viscosity, we mean the rate at which changes in the risk factor will influence the outcome. For instance, LDL cholesterol is likely to have high viscosity for CHD risk, as atherosclerosis is a chronic condition resulting from long-term exposure to LDL cholesterol. Short-term interventions in LDL cholesterol would not be expected to lead to immediate reduction in CHD risk. Indeed, increased time of exposure to LDL cholesterol–lowering drugs is associated with a greater reduction in CHD risk per 1-mmol/L change in LDL cholesterol (89), with Mendelian randomization estimates around two to three times greater in magnitude than even estimates of risk reduction from randomized trials with five-year median follow-up (17). By contrast, blood pressure may have lower viscosity as a risk factor, because although long-term exposure to high blood pressure does lead to arterial stenosis, current blood pressure is likely to be an important risk factor influencing CHD risk.

This life-course perspective for genetic associations has positive consequences for Mendelian randomization, as it means that genetic associations with disease outcomes may be greater than one would expect from observational research, and hence the power to detect a causal relationship may be greater than expected. However, it also means that causal estimates from Mendelian randomization are not realistic guides to the impact of intervening on a risk factor in practice and may be overly optimistic in general. Further research is needed to judge the similarity of observational epidemiological, clinical trial, and Mendelian randomization estimates in cases where all are available.

## 6 Additional Statistical Issues in Mendelian Randomization

A Mendelian randomization investigation has two related aims: first, to assess whether a risk factor has a causal effect on an outcome, and second, to estimate the magnitude of that causal effect. The first aim is usually the primary one, with estimation being a secondary aim. As discussed above, there are several reasons why a causal estimate obtained from Mendelian randomization is not always a reliable guide to the expected impact of intervening on the same risk factor in practice. In discussing statistical issues that may affect Mendelian randomization investigations beyond those related to invalid instruments, we focus on biases that affect not only estimates but also the validity of causal inferences, as these will lead to a greater-than-expected rate of false positive findings from applied investigations. Here, we focus on sample overlap and collider bias.

### 6.1 Weak Instrument Bias and Sample Overlap

If the genetic associations with the risk factor and those with the outcome are estimated in separate samples of the population, then the estimates will be independent. However, in practice, many large international genetics consortia include the same studies and hence have participants in common (71). This means that if genetic associations with the risk factor are overestimated, then associations with the outcome will also tend to be overestimated (assuming that the risk factor and outcome are positively correlated). This leads to weak instrument bias, a version of the winner’s curse in IV analysis (26). In a two-sample analysis, independence between the association estimates means that weak instrument bias is in the direction of the null and does not lead to inflated type 1 error rates. By contrast, in a one-sample analysis, bias is in the direction of the confounded association and can lead to false positive findings (78). When there is some overlap between the two data sets, bias is proportional to the degree of overlap (21). A weakness of summarized data is that it is often not possible to determine the degree of sample overlap or to correct for sample overlap by omitting individuals from the calculation of genetic association estimates or by performing cross-validation (27).

### 6.2 Collider Bias

A collider is a variable that is a common effect of two variables (that is, it is causally downstream of the two variables) (35). Even if the two variables are unrelated (they are marginally independent), they will typically be related when conditioning on the collider (conditionally dependent). For example, suppose that ear infections and throat infections are independent events. However, conditional on an individual attending an ear, nose, and throat clinic, ear infections and throat infections are conditionally dependent events—if an individual does not have an ear infection but attends such a clinic, then it is more likely that the individual has a throat infection (77). Conditioning on a collider between an IV and confounder can induce an association between the two even if they are marginally independent. This can lead to a valid IV becoming invalid and hence to an association between the IV and the outcome in the absence of a causal effect of the risk factor on the outcome. Intuitively speaking, even if a genetic variant can be treated as if it is randomly distributed in the general population, it may not be randomly distributed in a subset of the general population (in particular, in the sample population under investigation).

Collider bias can result from differential selection into the sample population (56). For example, if the sample is preferentially selected with respect to the risk factor or the outcome, then selection bias (a form of collider bias) would occur (both the risk factor and the outcome are common effects of the genetic variant and confounders, and hence both are colliders for these two variables). Collider bias may also result from differential survival. If the risk factor affects survival, then selection bias would also occur (95). This is particularly likely for Mendelian randomization investigations of diseases of old age (75). It is also relevant for investigations of disease progression, as in order to be included in an analysis of disease progression, one must have the disease in the first place. A further situation where collider bias may occur is when the population under investigation is stratified, in particular if the stratifying variable is downstream of the risk factor or outcome. A Mendelian randomization analysis of the causal effect of BMI on breast cancer progression showed that bias would occur if BMI were a cause of breast cancer risk (51). However, the magnitude of the selection effect required to lead to substantial type 1 error inflation was greater than would be plausibly expected, indicating that findings were likely to be robust to collider bias. A further weakness of summarized data is that the analyst has no control over how the data were collected, which studies were included in the case of consortium data, or what adjustment has been made in calculating the genetic association estimates. Further research is needed to ascertain the extent to which collider bias is likely to be a practically relevant issue in Mendelian randomization investigations.

## 7 Conclusion

This review has focused on answering two questions: When can Mendelian randomization be used to make reliable causal inferences, and when can it be used to make relevant causal inferences? As for the first question, the most reliable causal inferences from Mendelian randomization will always come from monogenic analyses (candidate gene Mendelian randomization), where the genetic variant has a plausible biological link with the risk factor of interest. There are some issues with these analyses, such as the problem of multiple testing. Additionally, the lessons of the candidate gene era—the need for replication and stringent *p*-value thresholds (although perhaps not as strict as a genome-wide threshold, since the number of comparisons is typically far lower)—must be learned. While polygenic Mendelian randomization analyses have inherent weaknesses, as the IV assumptions are unlikely to hold for all genetic predictors of a given risk factor, several methods are available for interrogating the robustness of findings. Although the inverse-variance weighted method will generally be the primary analysis method, all polygenic Mendelian randomization investigations should at minimum assess the heterogeneity between causal estimates from different variants, including a visual assessment via a scatter plot. Median-based and mode-based methods are useful for determining whether a causal effect is evidenced by the majority of genetic variants and for detecting multiple causal mechanisms within the genetic variants. In some cases, despite its decreased power, the MR-Egger method will also be relevant for detecting pleiotropy (via the intercept test) and for providing another estimate of the causal effect under a different set of assumptions. In other scenarios, such as when there are influential points in the regression model or when pleiotropic effects of multiple variants are likely to act via the same confounder, the MR-Egger method is less appropriate. Other approaches, such as multivariable Mendelian randomization and interactions and subsetting, will be useful in some situations. Cases in which multiple methods that make different assumptions lead to the same causal conclusion should be given more evidential weight in favor of a causal relationship.

As for the second question, while Mendelian randomization addresses a relevant causal question (that of the long-term effect of elevated levels of the risk factor on the outcome), it does not answer all relevant causal questions. In addition, particularly for highly viscous risk factors, causal estimates and even causal inferences may be misleading guides to the impact of interventions on the same risk factor in applied practice. While there is still much to learn from the emerging biobank era of data sets whose main distinguishing feature is their sheer size, there are also many biologically relevant questions about timings and mechanisms of causal effects that will require clever epidemiological designs as well as clever statistical analyses—not all causal questions will be answerable simply by increasing the sample size of cross-sectional data sets.

Future methodological questions to be determined include evaluating and comparing the various robust methods for Mendelian randomization, both those included in this review and further proposed methods (2, 42, 64, 76, 90, 96). A related question is how to take advantage of genome-wide genetic data, and even whether extensive genetic data will ever be useful for making reliable causal inferences. An additional area is how large-scale data on multiple biomarkers and multiple layers of -omics data can be used in Mendelian randomization investigations, particularly to learn about complex etiological networks and causal mechanisms.

We look forward to the further development of genetic knowledge, statistical methodology, and epidemiological data sets—active communication and close collaboration between these fields have been defining features of Mendelian randomization research so far. We have every confidence that this will continue.

## Supplementary Material

Supplementary information
