# Supplementary material for: Inferring Causal Relationships Between Risk Factors and Outcomes from Genome-Wide Association Study Data
Source: Annu Rev Genomics Hum Genet. Author manuscript; Available in PMC 2019 Apr 24. (PMC6481551; doi:10.1146/annurev-genom-083117-021731)
Supplement: Supplementary information [file NIHMS82522-supplement-Supplementary_information.pdf]

Supplemental Material: Annu. Rev. Genom. Hum. Genet. 2018. 19:303–27
https://doi.org/10.1146/annurev-genom-083117-021731
Inferring Causal Relationships Between Risk Factors and Outcomes from Genome-Wide Association Study Data
Burgess, Foley, and Zuber

Supplemental Appendix

Software code

We assume that summarized data are available on genetic associations with the risk factor (beta-coefficients `betaXG` and standard errors `sebetaXG`) and the outcome (beta-coefficients `betaYG` and standard errors `sebetaYG`). Example data are provided on genetic associations with C-reactive protein (log-transformed) and coronary heart disease risk (log odds ratios). We assume that all genetic variants are uncorrelated (in linkage equilibrium) – in reality, the genetic variants are all in different gene regions, so this assumption is reasonable. We provide both the primary code to perform the analysis, and the implementation using the *MendelianRandomization* package.

```
    #
    # data entry
    #
betaXG  =c(0.160, 0.236, 0.149, 0.09, 0.079, 0.072, 0.047, 0.05, 0.069,
  0.039, 0.088, 0.032, 0.104, 0.045, 0.054, 0.032, 0.032)
betaYG  =c(0.0237903, -0.1121942, -0.0711906, -0.030848, 0.0479207, 0.0238895,
  0.005528, -0.0327605, 0.0214852, -0.0387675, -0.0304042, -0.0082261,
  0.0246432, 0.0148795, -0.0498487, 0.0155667, 0.0242003)
sebetaXG=c(0.006, 0.009, 0.006, 0.005, 0.005, 0.005, 0.006, 0.006, 0.011,
  0.006, 0.015, 0.006, 0.015, 0.007, 0.009, 0.006, 0.007)
sebetaYG=c(0.0149064, 0.0303084, 0.0150552, 0.0148339, 0.0143077, 0.0145478,
  0.0160765, 0.0140347, 0.0255237, 0.0139256, 0.0441698, 0.0162031,
  0.0444987, 0.016674, 0.0220043, 0.018098, 0.0219547)
    #
    # install R package
    #
install.packages("MendelianRandomization")
library(MendelianRandomization)
    #
    # Inverse-variance weighted method
    #
betaIVW             = summary(lm(betaYG~betaXG-1, weights=sebetaYG^-2))$coef[1]
sebetaIVW.fixed     = summary(lm(betaYG~betaXG-1, weights=sebetaYG^-2))$coef[1,2]/
                       summary(lm(betaYG~betaXG-1, weights=sebetaYG^-2))$sigma
sebetaIVW.random    = summary(lm(betaYG~betaXG-1, weights=sebetaYG^-2))$coef[1,2]/
                   min(summary(lm(betaYG~betaXG-1, weights=sebetaYG^-2))$sigma,1)
MRpackage.IVW       = mr_ivw(mr_input(betaXG, sebetaXG, betaYG, sebetaYG))
    #
    # MR-Egger method
    #
betaYG = betaYG*sign(betaXG); betaXG = abs(betaXG)
betaEGGER           = summary(lm(betaYG~betaXG, weights=sebetaYG^-2))$coef[2,1]
sebetaEGGER.random  = summary(lm(betaYG~betaXG, weights=sebetaYG^-2))$coef[2,2]/
                   min(summary(lm(betaYG~betaXG, weights=sebetaYG^-2))$sigma, 1)
MRpackage.Egger     = mr_egger(mr_input(betaXG, sebetaXG, betaYG, sebetaYG))
    #
    # Median methods
    #
weighted.median <- function(betaIV.in, weights.in) {
  betaIV.order  = betaIV.in[order(betaIV.in)]
  weights.order = weights.in[order(betaIV.in)]
  weights.sum   = cumsum(weights.order)-0.5*weights.order
  weights.sum   = weights.sum/sum(weights.order)
        below   = max(which(weights.sum<0.5))
  weighted.est  = betaIV.order[below] + (betaIV.order[below+1]-betaIV.order[below])*
                 (0.5-weights.sum[below])/(weights.sum[below+1]-weights.sum[below])
  return(weighted.est) }
    #
weighted.median.boot = function(betaXG.in, betaYG.in, sebetaXG.in, sebetaYG.in, weights.in){
med = NULL
for(i in 1:1000){
 betaXG.boot = rnorm(length(betaXG.in), mean=betaXG.in, sd=sebetaXG.in)
 betaYG.boot = rnorm(length(betaYG.in), mean=betaYG.in, sd=sebetaYG.in)
 betaIV.boot  = betaYG.boot/betaXG.boot
```

```
 med[i] = weighted.median(betaIV.boot, weights.in)
 }
return(sd(med)) }
    #
betaIV              = betaYG/betaXG
weights             = rep(1, length(betaXG))
betaSIMPLEMED       = weighted.median(betaIV, weights)
sebetaSIMPLEMED     = weighted.median.boot(betaXG, betaYG, sebetaXG, sebetaYG, weights)
MRpackage.smedian   = mr_median(mr_input(betaXG, sebetaXG, betaYG, sebetaYG), weighting = "simple")
    #
betaIV              = betaYG/betaXG
weights             = (sebetaYG/betaXG)^-2
betaWEIGHTEDMED     = weighted.median(betaIV, weights)
sebetaWEIGHTEDMED   = weighted.median.boot(betaXG, betaYG, sebetaXG, sebetaYG, weights)
MRpackage.wmedian   = mr_median(mr_input(betaXG, sebetaXG, betaYG, sebetaYG), weighting = "weighted")
    #
    # Robust regression (MM-estimation with Tukey's bisquare objective function)
    #
library(robustbase)
betaIVWrobust       = summary(lmrob(betaYG~betaXG-1, weights=sebetaYG^-2, k.max=500))$coef[1] # change
sebetaIVWrob.random = summary(lmrob(betaYG~betaXG-1, weights=sebetaYG^-2, k.max=500))$coef[1,2]/
                  min(summary(lmrob(betaYG~betaXG-1, weights=sebetaYG^-2, k.max=500))$sigma,1)
MRpackage.IVWrobust = mr_ivw(mr_input(betaXG, sebetaXG, betaYG, sebetaYG), robust=TRUE)
```

```
    #
    # Modal estimation
    # (see reference 30 for more explanation)
    #
library(matrixStats);
library(iterpc);
library(Matrix);
library(stats);
library(optimbase);
  #
model.prior = function(model.size, N.obs, prob.valid.inst){
  pr = (prob.valid.inst^model.size)*(1-prob.valid.inst)^(N.obs-model.size)
  return(pr)
}
  #
het.weight = function(prob.valid.inst, bx, by, byse){
  J = length(by);
  theta.est = by/bx;
  theta.se = abs(byse/bx);
  tmp.1 = by/byse;
  tmp.2 = bx/byse;
  theta.se.sq = theta.se^2;
  log.theta.se = log(theta.se);
  est  = seest = vector("numeric", 2^J-1);
  het.weight = vector("numeric", 2^J-1);
   #
  count = 0;
 for(n in 1:J){
    perms = choose(J,n);
    inc = sparseMatrix(i=as.vector(t(replicate(n,1:perms))),
                       j=as.vector(t(getall(iterpc(J,n,c(1:J))))),
                       x=1, dims = c(perms,J));
    # sparse binary inclusion matrix
    # 1 denotes an instrument is included in the model
    # each row represents a particular model
    est.sum = inc%*%(theta.est/theta.se.sq);
    recip.var.ivw = inc%*%(1/theta.se.sq);
    est.ivw = est.sum/recip.var.ivw;
    est[(count+1):(count+perms)] = est.ivw;
  if(n>1){
     tmp = t(replicate(J, as.vector(est.ivw)));
   if(n<J){
     psi.hat = sqrt((1/(n-1))*rowSums(t(t(inc)*(tmp.1^2 - 2*tmp*(tmp.1*tmp.2) +
                    (tmp^2)*(tmp.2^2)))))
           }
      else{
        psi.hat = sqrt((1/(n-1))*sum(tmp.1^2 - 2*tmp*(tmp.1*tmp.2) +
                    (tmp^2)*(tmp.2^2)));
           }
      psi.hat[which(psi.hat<1)] = 1;
      seest[(count+1):(count+perms)] = psi.hat/sqrt(recip.var.ivw);
          }
  else if(n==1){
      seest[(count+1):(count+perms)] = inc%*%theta.se;
                }
     #
  if(n>1){
      het.exponent = rowSums(inc*t(t(t(t(inc)*theta.est) -
            as.vector(est.ivw))^2/theta.se.sq));
      het.weight[(count+1):(count+perms)] =
                 exp(-(inc%*%(log.theta.se)+0.5*het.exponent))*
                 model.prior(n,J,prob.valid.inst);
          }
    count = count+perms;
  }  # ends for loop
  newlist = list(het.weight, est, seest);
  return(newlist)
}
   #
results = het.weight(0.5, betaXG, betaYG, sebetaYG);
het.weight = results[[1]];
het.weight.norm = het.weight/sum(het.weight);
```

```
   # normalized heterogeneity-penalized weights
betaMODE   = results[[2]];
sebetaMODE = results[[3]];
   #
sumlik=NULL
grid.increment = 1e-3; grid.start = -1; grid.end = 1;
point = matrix(seq(grid.start, grid.end, grid.increment), ncol = 1);
   #
l = length(het.weight.norm);
  #
sumlik = vapply(point,function(i){sum(het.weight.norm*dnorm(rep(i,l), betaMODE, sebetaMODE))}, 1);
   # calculates the likelihood at a range of values from -1 to +1
   # if the causal effect may be outside of this range,
   #  then this range of values will need to be expanded
whichin = which(2*log(sumlik)>(2*max(log(sumlik))-qchisq(0.95, df=1)));
   # provides an index of estimate values in the 95% confidence interval
estimate = -1.001+0.001*which.max(log(sumlik));
   # modal estimate
ifelse(sum(diff(whichin)!=1)==0, "Single range", "Multiple ranges");
   # returns "Single range" if the 95% CI is a single range of values
   # returns "Multiple ranges" otherwise
lowerCI    = -1.001+0.001*whichin[1];
upperCI    = -1.001+0.001*whichin[length(whichin)];
   # lower and upper confidence interval limits (assuming single range)
fullCI     = -1.001+0.001*whichin;
pval.mode  = pchisq(2*(log(max(sumlik))-log(sumlik[1001])), df=1, lower.tail=FALSE)
```

```
   #
   # Simple plots
   #
   # 1. Scatter plot (gene--outcome associations against gene--risk factor associations)
   #                 (lines represent 95\% confidence intervals)
betaYG = betaYG*sign(betaXG); betaXG = abs(betaXG)
plot(betaXG, betaYG, xlim=c(min(betaXG-2*sebetaXG, 0), max(betaXG+2*sebetaXG)),
 ylim=c(min(betaYG-2*sebetaYG, 0), max(betaYG+2*sebetaYG, 0)))
for (j in 1:length(betaXG)) {
 lines(c(betaXG[j],betaXG[j]), c(betaYG[j]-1.96*sebetaYG[j], betaYG[j]+1.96*sebetaYG[j]))
 lines(c(betaXG[j]-1.96*sebetaXG[j],betaXG[j]+1.96*sebetaXG[j]), c(betaYG[j], betaYG[j]))
 }
abline(h=0, lwd=1); abline(v=0, lwd=1)
   #
   # 2. Funnel plot (measure of precision of IV estimate -- specifically the reciprocal of the standard error
   #                 versus the IV estimate)
plot(betaYG/betaXG, betaXG/sebetaYG, xlim=c(min((betaYG-2*sebetaYG)/betaXG),
  max((betaYG+2*sebetaYG)/betaXG)), ylim=c(0, max(betaXG/sebetaYG)))
for (j in 1:length(betaXG)) {
 lines(c((betaYG[j]-1.96*sebetaYG[j])/betaXG[j],  (betaYG[j]+1.96*sebetaYG[j])/betaXG[j]),
  c(betaXG[j]/sebetaYG[j], betaXG[j]/sebetaYG[j]))
 }
abline(h=0, lwd=1); abline(v=0, lwd=1)
```

Additional details of applied examples

C reactive protein and coronary heart disease: All variants were previously demonstrated to be associated with CRP levels at a genome-wide level of significance by Dehghan et al. [Meta-analysis of genome-wide association studies in >80 000 subjects identifies multiple loci for C-reactive protein levels. Circulation 2011; 123(7):731–738].

rsid	Nearest gene	Effect allele	Association with CRP Beta (SE)	Association with CHD risk Beta (SE)	Causal estimate Estimate (SE)
rs2794520	*CRP*	C	0.160 (0.006)	0.024 (0.015)	0.149 (0.093)
rs4420638	*APOC1*	A	0.236 (0.009)	-0.112 (0.030)	-0.475 (0.128)
rs1183910	*HNF1A*	G	0.149 (0.006)	-0.071 (0.015)	-0.478 (0.101)
rs4420065	*LEPR*	C	0.090 (0.005)	-0.031 (0.015)	-0.343 (0.165)
rs4129267	*IL6R*	C	0.079 (0.005)	0.048 (0.014)	0.607 (0.181)
rs1260326	*GCKR*	T	0.072 (0.005)	0.024 (0.015)	0.332 (0.202)
rs12239046	*NLRP3*	C	0.047 (0.006)	0.006 (0.016)	0.118 (0.342)
rs6734238	*IL1F10*	G	0.050 (0.006)	-0.033 (0.014)	-0.655 (0.281)
rs9987289	*PPP1R3B*	A	0.069 (0.011)	0.021 (0.026)	0.311 (0.370)
rs10745954	*ASCL1*	A	0.039 (0.006)	-0.039 (0.014)	-0.994 (0.357)
rs1800961	*HNF4A*	C	0.088 (0.015)	-0.030 (0.044)	-0.346 (0.502)
rs340029	*RORA*	T	0.032 (0.006)	-0.008 (0.016)	-0.257 (0.506)
rs10521222	*SALL1*	C	0.104 (0.015)	0.025 (0.044)	0.237 (0.428)
rs12037222	*PABPC4*	A	0.045 (0.007)	0.015 (0.017)	0.331 (0.371)
rs13233571	*BCL7B*	C	0.054 (0.009)	-0.050 (0.022)	-0.923 (0.407)
rs2836878	*PSMG1*	G	0.032 (0.006)	0.016 (0.018)	0.486 (0.566)
rs4903031	*RGS6*	G	0.032 (0.007)	0.024 (0.022)	0.756 (0.686)

Table 1: Details of genetic variants, beta-coefficients (standard errors, SE) for associations with C-reactive protein (CRP, log-transformed) and with coronary heart disease (CHD) risk, and causal effect estimates (log odds ratios for CHD per unit increase in log-transformed CRP) for 17 genome-wide significant variants.

LDL cholesterol: The example analysis using genetic variants specifically linked with LDL cholesterol includes 8 genetic variants. These variants are in gene regions: *HMGCR* (proxy for statin treatment), *PCSK9* (proxy for PCSK9 inhibition), *NPC1L1* (proxy for ezetimibe), *APOB* (encodes biologically relevant apolipoprotein B), *ABCG5/G8* (bile acid sequestrant), *SORT1* (antisense oligonucleotide RNA inhibitor targeting this pathway currently under development), *APOE* (encodes biologically relevant apolipoprotein E), and *LDLR* (encodes biologically relevant LDL receptor). The specific choice of variant in each gene region to include in the analysis was based on the lead variant from the Global Lipids Genetic Consortium's 2010 analysis.

rsid	Nearest gene	Effect allele	Association with LDL-c Beta (SE)	Association with CHD risk Beta (SE)	Causal estimate Estimate (SE)
rs12916	*HMGCR*	C	2.729 (0.360)	0.036 (0.009)	0.566 (0.150)
rs2479409	*PCSK9*	G	1.354 (0.366)	0.029 (0.010)	0.556 (0.200)
rs2072183	*NPC1L1*	C	1.377 (0.425)	0.014 (0.012)	0.451 (0.394)
rs1367117	*APOB*	A	3.544 (0.374)	0.041 (0.011)	0.393 (0.101)
rs4299376	*ABCG5/G8*	G	2.458 (0.375)	0.051 (0.010)	0.714 (0.147)
rs629301	*SORT1*	T	4.654 (0.437)	0.101 (0.011)	0.694 (0.078)
rs4420638	*APOE*	G	6.003 (0.461)	0.092 (0.014)	0.498 (0.076)
rs6511720	*LDLR*	G	8.583 (0.578)	0.125 (0.017)	0.693 (0.094)

Table 2: Details of genetic variants, beta-coefficients (standard errors, SE) for associations with LDL cholesterol (mg/dL) and with coronary heart disease (CHD) risk, and causal effect estimates (log odds ratios for CAD per 1 mmol/L increase in LDL cholesterol) for 8 genetic variants.
